# Children's perceptions of environment and health in two Scottish neighbourhoods

**DOI:** 10.1016/j.socscimed.2021.114186

**Published:** 2021-08

**Authors:** Niamh K. Shortt, Chris Ross

**Affiliations:** a Centre for Research on Environment Society and Health, School of GeoSciences, University of Edinburgh, Scotland, EH8 9XP, United Kingdom; b Children in Scotland, Edinburgh, Scotland, EH12 5EZ, United Kingdom

**Keywords:** Children's health, Place-based stigma, Rights of the child, Social exclusion, Health inequalities

## Abstract

This article explores children's understanding of the role that neighbourhood plays in their health and well-being. Whilst evidence exists on the relationship between the environment and children's health, we have little knowledge of this from the perspective of children themselves. Children's experiences are all too frequently researched through the eyes of adults. Following a Rights of the Child framework, respecting children's views and giving them due weight, this paper reports from a project that worked with children from two relatively deprived urban neighbourhoods in Scotland. Using this framework, the children themselves were the researchers who designed the themes, decided upon the methods, conducted the research and analysed the resulting data. Using focus groups, visual mapping and community walks the children explored their local neighbourhoods and the findings reveal features of the environment that the children perceive as important for their health and well-being. The children selected three themes to explore in the research: safety, littering, and family and friends, through which they elicit their experiences, feelings and attitudes towards the environment and their well-being. The paper reveals that not only do the children have a deep understanding of the link between environment and health, but that they also understand how aspects of disadvantage, including place-based stigma, can limit their social participation and inclusion in society. We conclude with recommendations made by the children themselves, ranging from access to affordable activities, improved open spaces, ‘support not stigma’ and the need to be heard in local decision making.

## Highlights

• Children understand the relationship between neighbourhoods and health.
• Three key themes were highlighted: safety, littering and family and friends.
• Place-based stigma and exclusion may limit children's social participation.
• The ‘in place’ experiences of children are important for health research.
• Children can play a role in creating places to improve their health and well-being.

## 1 Background

Research has demonstrated an association between features of the neighbourhood and population health, with a large body of research exploring these associations and the mechanisms that drive them. This work, framed within a broader determinants of health perspective, addresses macro level political, social, and economic policies that lead to unequal neighbourhoods. In recent years, there has been a move towards involving residents in an exploration of these broader determinants, for example through citizen juries. Whilst much of this has focussed on adults, research has begun to explore how children experience their environments and how they understand the association between neighbourhoods and health. Researchers recognise that “research about children's lives is … essential if policies and programmes are to become more responsive and relevant to their concerns and needs” (Boyden and Ennew, 1997, p. 10). In this paper we explore children's understanding of the role that neighbourhood plays in their health and well-being. We focus on children residing in high poverty areas, reflecting a particular interest in understanding their experiences. The children were engaged as researchers and active participants to ensure that their voices are heard and that their insights have potential to influence policy and practice in creating child-friendly neighbourhoods.

It has been suggested in the literature that there may not be a singular area effect on health (Macintyre et al., 2002). Instead, features of local areas may have differential impacts upon various population groups. Local neighbourhoods will inevitably have greater influence on the health of populations who are more mobility constrained, such as children. The health and development of children is shaped by a plethora of factors, with family and friendship networks being of critical importance (Carter et al., 2007). Whilst evidence suggests that such proximal influences are critical, wider environments may also have a degree of influence, with Villanueva et al. stating that “children develop in multiple contexts including the family, peer group, and broader social and physical environments” (2016, p. 10). Younger age groups spend a significant amount of time in their local neighbourhoods, either on their own, with peer groups or with family. Neighbourhood characteristics that may impact upon the health and well-being of children and adolescents include air pollution (associated with decreased lung function (Salvi, 2007)), urban sprawl, levels of violent crime and low levels of green space (associated with physical activity (Gomez et al., 2004)), the food environment (associated with overweight and obesity (Osei-Assibey et al., 2012)), the built environment (related to physical activity (Smith et al., 2017)) area-level deprivation (associated with general health (Gomez et al., 2004; Picavet et al., 2016)) and social capital (associated with well-being (Eriksson and Dahlblom, 2020)).

Whilst we are building up a quantitative understanding of the importance of the environment for health and well-being, there have been calls to engage with a welfare research paradigm, one that addresses the importance of a lay perspective. Macintyre and colleagues argued that the quantitative evidence base *‘may fail to capture more subtle and intangible features of local environments which may nevertheless influence people's lives and health’* (Macintyre et al., 1993, p. 230). Responding to this, there is now a growing evidence base of research that has moved towards a ‘reconceptualisation of place’ (Popay et al., 1998, p. 635), bringing individuals into the analysis and emphasising the importance of this lay perspective (see for example Stead et al., 2001; Airey, 2003; Robertson, 2006; Thomas, 2016). Such a perspective recognises the need to bring a diverse range of stakeholders into the knowledge process. One stakeholder group, often excluded in this process, are children. As such, our understanding of children's perceptions of environmental risks and benefits is less well developed, resulting in a smaller evidence base for place-based interventions to support children. To address this, researchers have begun to engage with children and young people as active participants, particularly within the sub-discipline of children's geographies. Rather than objectifying children in the research process and performing research ‘on’ children, researchers have begun to involve children as active agents in child-centred methods (Ergler et al., 2021). Such an approach is centred on the idea that children are “closer to understanding their world and how to improve their well-being” than adult researchers (Ergler in Coles and Millman, 2013, p. 187).

This child centred approach is enshrined in the United Nations Convention on the Rights of the Child, the most widely ratified global human rights treaty. The treaty consists of 54 articles, including Article 12, the ‘Right to be Heard’ stating that: *“Parties shall assure to the child who is capable of forming his or her own views the right to express those views freely in all matters affecting the child, the views of the child being given due weight in accordance with age and maturity of the child”* (UN Commission on Human Rights, 1990). This increasing recognition of children's rights emphasises the importance of incorporating children not just as participants, but as co-researchers, designing and leading research into areas that have an impact upon them. Methods of engaging children have been theorised in participation frameworks, some adopting hierarchical notions, such as Hart's (1992) ‘Ladder of Children's Participation’ (built on Arnstein's 1969 model) (Hart, 1992), and others removing this hierarchy of engagement, such as Lundy's ‘Voice’ model (Lundy, 2007). The ‘Voice’ model, developed to help conceptualise Article 12, consists of four factors that enable a child's right to participate in decision making: space (children are afforded the opportunity to express their view in a safe and inclusive space), voice (children are facilitated to express their view), audience (the view should be listened to) and influence (the view should be acted upon) (Kennan et al., 2019). Reflecting Article 12, the first two factors afford children and young people the right to express views, whilst the latter two, the right to have views given due weight.

Within the environment and health field, research conducted with children regarding the neighbourhood has been varied. Topics have ranged from a focus on traffic (Mullan, 2003), neighbourhood trustworthiness and safety (Meltzer et al., 2007), and social capital (Morrow, 2000), through to an exploration of harmful environmental factors and environment borne disease (Pluhar et al., 2009). In much of this work, the features of the neighbourhood that have been explored have been chosen by adults and the research has been adult led. It could be argued that within this work the children have been given space, a voice and audience, reflecting Lundy (2007). Their views, whilst recorded and discussed, have however been measured in response to adult defined concerns. As a result, we know relatively little of what the children and young people themselves perceive to be important neighbourhood level drivers of health. Allowing children and young people to define the selection of neighbourhood features for inquiry creates the broader conditions to ensure that their ideas have influence, and that their views are given due weight at all stages of the research.

Some research on environment and health has allowed children and young people to guide theme selection and to describe aspects of the environment that either relate to health more broadly, or to specific elements of health and well-being. An example of this from Irwin and colleagues (2007) explored children's perspectives on their local contexts and perceived connections between their living conditions and health outcomes. Working in a ‘neighbourhood associated with vulnerability’ (p. 353), the children reported what may be more typically associated with health, for example the need to eat healthy food and be physically active. Children also offered perspectives on the neighbourhood, including having few opportunities to play and psychosocial stresses linked to physical safety (Irwin et al., 2007). Exploring city spaces, Carrol et al. (2019) asked children how neighbourhood features impacted upon their mobility, social interactions and recreational opportunities. The children in this study discussed what makes a ‘good’ environment, alongside features of the environment that may restrict their mobility, such as safety and traffic.

Assessing children's understanding of health and neighbourhood influences on health is challenging. Allowing children to shape the research agenda, be part of the research and perform as researchers adds additional layers of complexity. Doing so, however, can enable children to be full stakeholders in their neighbourhoods and to be agents of change. Research has demonstrated that place is important for health and well-being, but as Jones and Moon argue, *‘seldom…* *does location itself play a real part in the analysis; it is the canvas on which events happen, but the nature of the locality and its role in structuring health status and health related behaviour is neglected’* (1993, p. 515). To address this relative neglect, we describe a project conducted with children and young people that explores the role that neighbourhood plays in structuring health and well-being from the perspective of those aged between 10 and 14. This project, based on the premise that ‘health is produced in everyday life’ (Bernard et al., 2007), aims to uncover the canvas on which these individual children act out their lives. In line with Lundy's ‘Voice’ model, we sought to implement all four elements, ensuring that the children could both express their views and that these views were given due weight. Reflecting this, the broad aims of the project were designed by adults, but as outlined further in the methods, the children themselves emerged as the young researchers who chose the themes to be explored, giving weight to their perceptions. These young researchers also collected data and analysed the findings. The research aimed to uncover how children living in more deprived neighbourhoods perceive their environments, and in turn, how features of their neighbourhoods contribute towards shaping the canvas upon which their health and well-being is produced.

## 2 Methods

This co-produced research with a children's charity, Children in Scotland, worked in partnership with 15 young researchers based in two schools, one in Dundee and one in Glasgow. As an organisation, Children in Scotland is committed to ensuring that their work supports and upholds the rights of the child. This ethos informed the methodology, a participative research approach, applied to the project. The project aimed to address two broad research questions:

1. How do community and place impact on health and wellbeing for children and young people?
2. How might this contribute to health inequalities between different areas?

To answer these questions from a child centred approach, we began the project by recruiting young researchers who would work with groups of children and young people (of similar ages) as participants. The young researchers facilitated focus groups and mapping exercises, conducted an ethnographic community walk around, and analysed the resulting data.

To begin, we selected two schools in different areas of Scotland. We chose to work with children in both a secondary school and a primary school. The age of children in primary school in Scotland ranges from 4 to 12 and in secondary school from 11 to 18. To be involved in the project, the children and young people had to be aged between 10 and 18. As our focus was on working with those attending schools in more deprived areas, we chose to focus on selecting schools located in areas that have higher rates of poverty than the national average. Schools in such areas were identified through existing relationships held by Children in Scotland. Contact was made with these schools to identify those that were willing to participate. Two schools were chosen: a primary school, based in a highly urbanised area of Glasgow, and a secondary school, situated in a more suburban area of Dundee.

Contact was made with the schools to recruit children and young people to act as young researchers. Schools were asked to identify eight pupils from one specific year group. To ensure diversity in our researchers, we asked the schools to consider students who would not normally get the opportunity to become involved in projects and to consider young people with additional support needs. We also asked the schools to consider the gender and ethnic representation of their eight selected students. To ensure parental support, the parents of those selected were informed and written consent was gathered from both the young researchers themselves and their parents. Risks were also discussed with the young researchers in advance, including issues related to child protection in case any of the participating children disclosed sensitive information. The young researchers were given a contact at Children in Scotland and staff were available to respond to any issues. In total, fifteen young researchers were recruited; eight from the primary school in Glasgow (Primary 6 at start of project and Primary 7 at completion, aged between 10 and 12) and seven from the secondary school in Dundee (S1 at start of project and S2 at completion aged between 11 and 13).

### 2.1 Capacity building

Before commencing with research design, we worked with the young researchers to explore their base knowledge on issues related to health and well-being. To identify key themes, a combination of visual prompts and case study presentations were used to stimulate discussion. Using MacIntyre et al.’s (1993) five broad types of socio-environmental influences on health (physical features, health environments, services, socio-cultural features and area reputation) a series of flash cards were designed to cover the key determinants of health and the role of place in health inequalities. These flash cards were shown to the young researchers and discussion followed. Had the young researchers identified other topics, these could have been explored, however this did not occur. We then used a number of case studies based on fictional characters that explored the day of a child in a community and discussed how the resources and their experiences locally might affect their health. Further discussions were held on inequalities, including the causes of inequalities, and again using visual prompts such as the Glasgow train line (McCartney, 2010) and Edinburgh tram line (Public Health Scotland, 2015) graphics, detailing how heath differs in different parts of the same city.

Following their discussions on place, health and inequalities, the young researchers were trained in research skills. Employing a range of games and role play activities, we worked with the young researchers to explore how they could ask questions, both to ensure open responses from their participants and to avoid asking leading questions. Using mix and match activities to sort information into themes, and a series of other games/activities, we developed the young researchers’ analytical skills to help them to identify relevant information and perform thematic analysis. During this session, we also explored bias and opinion and worked with the young researchers to understand how to try to reduce bias in their analysis and data collection.

Work to develop the young researchers’ skills in conducting research took place over one and a half days. This included one full day session and one half day refresher session before the focus groups. We also revisited this across the project at various point to refresh their skills and knowledge.

Following these discussions, the young researchers chose three neighbourhood themes that they identified as being important for health and well-being:

1. Safety
2. Littering
3. Family and Friends

### 2.2 Research design

At the outset, it was agreed that this project would apply a qualitative approach. Potential qualitative methods were identified and the advantages and disadvantages of each were discussed with the young researchers. The young researchers wanted to pursue a mixed methods approach and employ three broad methods: focus groups, visual methods, and ethnographic community walks.

The young researchers felt that focus groups would enable them to facilitate group discussions to identify areas of agreement or difference within the groups of participating children and young people. Participants in the focus groups were identified from the same school year group of the young researchers. Participants were identified by the participating schools based on factors including potential interest, willingness to participate and demographics of the year. Consent was sought to participate from the parents of participating children and young people. The focus groups were held during the school day and were facilitated by the young researchers. Members of staff from Children in Scotland were on hand to oversee the discussions, but these were entirely child-led. In total, 6 focus groups were delivered.

The young researchers identified a qualitative mapping exercise as a form of visual method, which was employed following the focus groups. Using school catchment maps, focus group participants were asked to highlight the places on the maps that they had referred to during the discussion. Using red (negative) and green (positive) stickers, participants were asked to identify areas on the maps where they felt safe or unsafe, areas that they visit with family and friends, areas where they would like to go, and areas where they would see a lot of litter or very little litter. The participants had 3 dots of each colour to use for each theme. These maps were then used by the young researchers to plan the ethnographic community walks.

In groups of 3–4 people (in addition to members of Children in Scotland staff for safety), the young researchers embarked on ethnographic walks in their local community. Walks took place over 4 days at each school, and lasted approximately 3–4 hours on each day.

Groups considered the different themes in turn; they each focussed on a research theme for one day to ensure all had the chance to engage with all areas of the research. During the walks, the young researchers paused for questions and discussion at sites that had been marked on the maps and highlighted in the focus groups. Notes were taken during the walks by the young researchers on themed sheets, where required adults wrote down notes for the young researchers. The young researchers also took photographs, pausing to capture discussion at these points. Each photo was given a number which was noted on the sheets. The location and key features of each photo were also noted. This information was used to match notes to photos.

### 2.3 Analysis and recommendations

The young researchers analysed the data collected from the focus groups, maps, and community walks. The young researchers were asked to consider how their findings could help them to answer the research questions, how the discussions of their neighbourhoods relate to health and well-being, and how the features of the neighbourhoods discussed may impact upon the lives of children and young people. Supporting adults completed the write up of the research, drawing directly on the work of the young researchers and using notes from the analysis sessions. Young researchers were offered the chance to support the write up of the report, but decided they did not wish to be involved. Following reflection of the findings, the young researchers identified recommendations that they would like to take forward from the research. These recommendations were based on discussion about what they had found out about their community and what they felt needed to change to support better health and wellbeing.

## 3 Results

In this section, we present the data from all methods employed by the young researchers. Focussing on the three research themes identified by them (safety, littering and friends and family), we present a mixture of interpretations from both the young researchers and the authors. While the young researchers did not rank the importance of any of these themes, safety did, however, dominate their discussions, with less focus on littering and family and friends.

### 3.1 Safety – “feel like everything is broken”

**Fig. 1:**
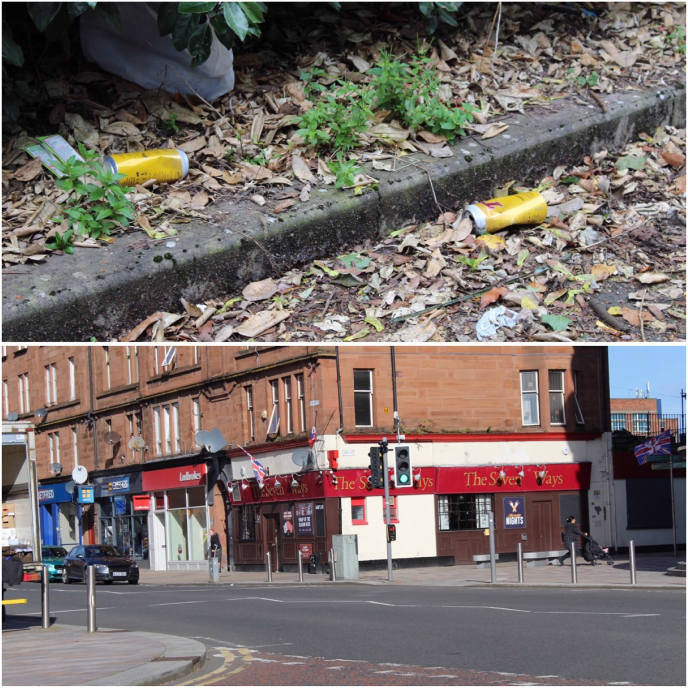
a Substance misuse debris and b High numbers of licensed premises in the neighbourhoods.

The research participants and young researchers in both areas highlighted issues related to substance misuse in their neighbourhoods, with related visual evidence making them feel less safe in their local environment. Substance misuse covered drugs (needles, pill packets and joints) and alcohol and cigarette use. The participants felt that the visual presence of related debris (Fig. 1a) and drug dealers meant that many of them avoided local parks and green spaces. During the community walks, the young researchers stopped in one area that was highlighted as unsafe in the mapping exercise. Whilst standing in the space, they queried why young people may feel unsafe in this space, with the response related to the high presence of bars (Fig. 1b). They stated that due to this, there were *“a lot of drunk people, they spit, worried about what they might do”*. They went on to say that *“drunk people do spooky stuff”* and *“you don't know what kind of person might come out. Some people might just have one drink. There should be a limit”*. The participants in our study recognised that adults may also be impacted by substance abuse in the area, with reference to potential abuse within the home: *“if you have an abusive partner, they come back drunk and start hitting you and your children”.*

The participants were fully aware of the impact that substance abuse would have on their health and well-being, both in terms of closing off spaces to them, but also recognising the impact upon social norms and behaviours. The participants highlighted the fact that many of them may be tempted to engage in such risky behaviours having witnessed them as ‘normal’ activities in their neighbourhoods. They did, however, understand that substance abuse was harmful for health, citing how it can damage development and cause early death, as well as poor mental health.

Beyond substance abuse, but related to safety, the participants discussed negative perceptions of crime in their neighbourhoods, and how both real and perceived crime may impact upon their health and well-being. They discussed their fears of gang violence and knife crime and the physical danger that may arise, but also the fear around the threat of violence and the stress and distrust this causes. One young person mentioned threats from strangers as children go about their daily lives. He evidenced this by saying “*my brother was playing football and strangers said they would bury him”*. During the walks, the young researchers also pointed out gang related graffiti on buildings, and in the focus groups spoke of how the presence of gangs and violence may mean that children and young people themselves get drawn in and begin to carry knives for personal protection.

People were discussed in relation to safety, in both a positive and negative sense. The participants spoke of how family members and friends can make them feel safe. People with community roles, such as teachers, the police, ‘the guy at the shop’ and the lollipop person were all identified as helping the children feel safe, secure and in turn, happy. The participants highlighted their positive relationships with these individuals, citing trust as a key feature in their extended relationships. One young person stated, *“it makes me feel safe because I'm getting protected”*. Not all adults were perceived as trustworthy; some also make the children and young people feel scared. They mentioned alleged paedophiles and *“weird, scary, strange people”* resulting in parents being afraid to let the children out on their own, particularly to certain areas such as parks. Such reflections are reminiscent of the findings in New Zealand where children's encounters with ‘weird’ people were reported (Witten et al., 2015).

**Fig. 2:**
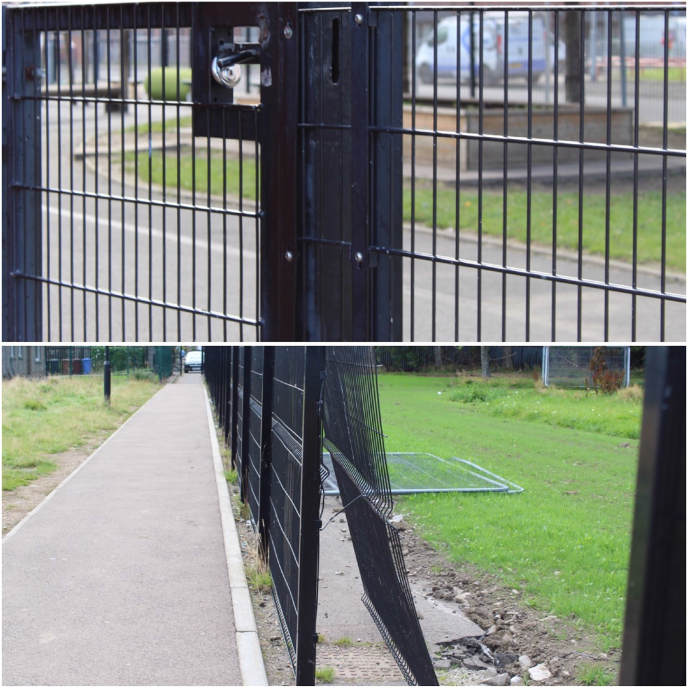
a Secure fence around school. b Broken fence around school.

The participants also related certain spaces to safety, with both schools and home highlighted as places where the children felt most safe. At home, this related to being together with family, where they felt secure, taken care of, and supported. The feeling of safety at school appeared to relate to both the reassuring presence of teachers but also the large wrought iron fences surrounding the schools (Fig. 2a). The participants discussed how the presence of the locked gates at schools make them feel safe and would stop unwelcome people entering and leaving. In a further image, we can see a broken part of the fence (Fig. 2b). The children paused here to say that this made them feel unsafe because *“people hang out there, people set a fire in the old school”.*

**Fig. 3:**
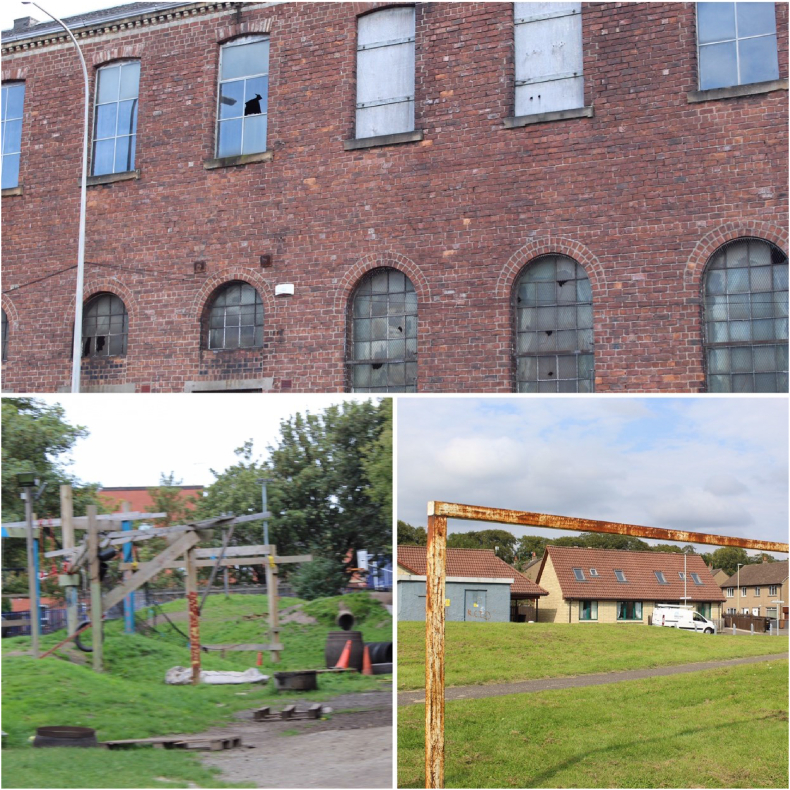
a An abandoned metal factory. b The adventure park. c Rusty play equipment.

On the walks, the young researchers paused at several abandoned buildings and discussed how such spaces make them feel. Not only did they mention feeling unsafe, but also that such spaces make it *“feel like everything is broken”,* that people *“just don't care”* about their neighbourhood and that such buildings are scary (Fig. 3). Derelict open spaces were lamented, with the participants recognising that such spaces *“could be made into an actual half decent park”*. Parks and open spaces were discussed as both safe and unsafe. One adventure park was explored on a community walk with the participants expressing their happiness that it is *“somewhere for children to actually play and it's free”*. This park was seen as positive, with staff members if children are hurt and a gate for safety (Fig. 3b). Others saw parks as dangerous, related to violence and substance misuse with old play equipment, for example rusty goals and broken play equipment (Fig. 3C), meaning that they are not used as play spaces.

Reflecting on inequalities, the young researchers on the walks paused in what they called *“the posh bit”*, a housing estate with ‘new’ and ‘expensive’ homes with ‘fences.’ They discussed how the area felt safe, relaxed and how kids there may have freedom as the area *“feels nice”* with trees, green spaces and *“wiggly roads and roundabouts”* to deter speeding traffic. They did, however, discuss how people living in this ‘posh’ area may look down on other areas.

### 3.2 Littering – “because people think where they live is a dumpsite”

**Fig. 4:**
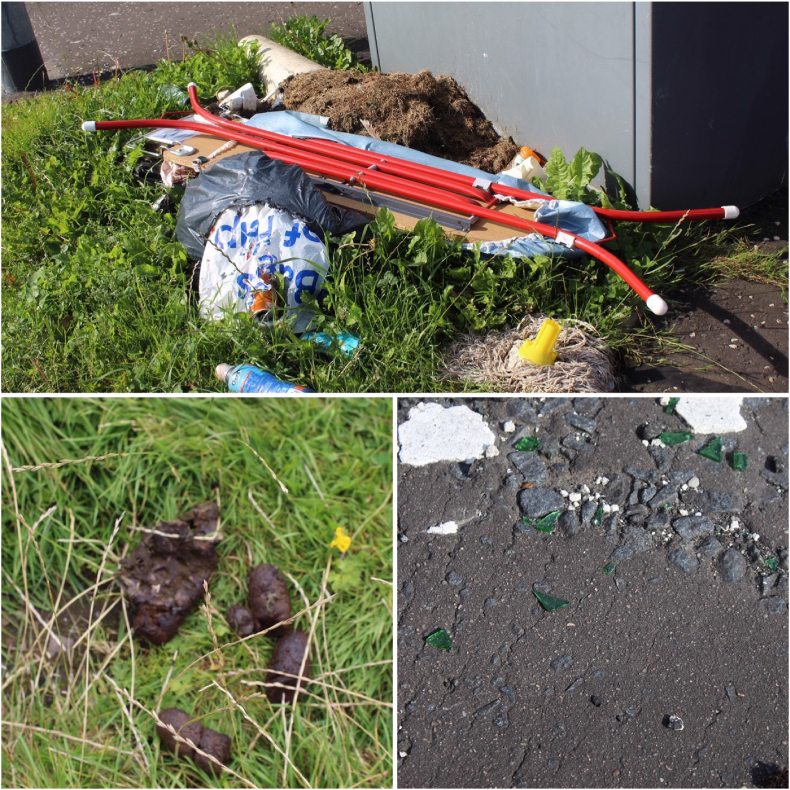
a Abandoned rubbish piles. b Dog waste. c Broken glass.

When considering littering and how it relates to health and well-being, the participants focussed on area reputation and inequalities. As when discussing safety, they expressed an awareness of the differences between areas, particularly between the ‘posh’ area and other areas. They observed the lack of bins in all neighbourhoods, but focussed on the fact that whilst there was only one bin in the ‘posh’ area, there was not much litter. The participants noted that in the more deprived area, the presence of large piles of rubbish *“makes it look like a bad area”*. This also made people have negative feelings towards their own neighbourhoods, making them feel “*horrible”*. In both areas, the young researchers paused on their walks to photograph rubbish piles (Fig. 4a), dog waste (Fig. 4b), broken glass (Fig. 4c), beer bottles and cans, and cracked and damaged pavements.

Much of the frustration around littering reflected the participants' worry for the environment. They spoke of how rubbish can hurt animals and how plastic waste can contribute to global warming; *“it's killing the planet,”* stated one of the participants. They made connections between the presence of litter and their mental well-being. They mentioned worries related to rubbish and the natural environment and made connections with their related anxiety regarding how others may feel about their area. Several participants mentioned how the heavy presence of litter made them want to move and made them feel embarrassed of where they live. During the walks, the young researchers stopped in places where they saw a lot of rubbish and stated that it made them feel that the area *“looks like it would be sketchy at night”*. It made them feel *“disgusted”* and *“disappointed”*, and as a result, they would want to avoid spending time in these areas. They discussed how those in charge appear not to care, as bins are not provided. As such, they didn't necessarily blame individuals for discarding small items of rubbish: *“who wants to carry rubbish for ages”*. Their views of larger informal dumping sites scattered throughout the neighbourhoods were however coupled with a narrative of laziness and a lack of civic responsibility. One young person summarised this by stating that people leave large amounts of rubbish on the side of the road *“because people think where they live is a dumpsite”.*

### 3.3 Family and friends – “you need money to come here”

When discussing this theme, the participants focussed on family and friend-based activities, particularly related to food, and shopping. Above all else, the children focussed on the home as a safe place where they feel nurtured and loved by immediate and extended family. They emphasised the importance of loving parents who care for them and family who make them feel safe. The home was frequently cited as a place where they can relax and escape all external pressures. It should however be noted that not all of the participants felt like this; there was some discussion of children who do not get along with their families and instead find support with their friends.

**Fig. 5:**
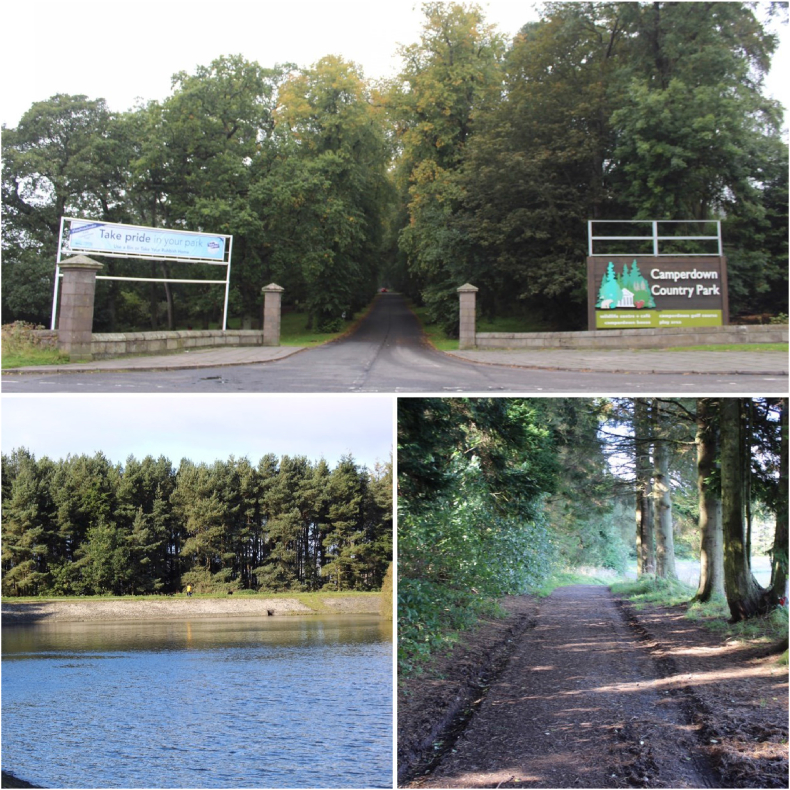
a An example of the Open Spaces photographed. b Entrance to Forest Park. C Reservoir.

Outside of the home, the participants discussed spaces that they visit with family and friends. On the walks, the young researchers paused at open spaces (Fig. 5a) including a forest park (Fig. 5b) and a reservoir (Fig. 5b). Discussion referring to escapism in these spaces was prominent; statements such as *“I don't have to think about much”* and *“I get away from what is happening”* reflect this. The participants were able to connect time spent in outdoor spaces with their family and friends to their health and well-being, discussing feelings of calmness, happiness, and fun. Whilst outdoor spaces are enjoyable for the participants, a common theme in their discussions was their inability to access these places with friends, without adult supervision. They noted that these open spaces are pleasant but that it is an “*effort to come”*, noting that there are rules about how people should act/behave, children need to be with parents, and as a result, these spaces can be ‘boring’.

**Fig. 6:**
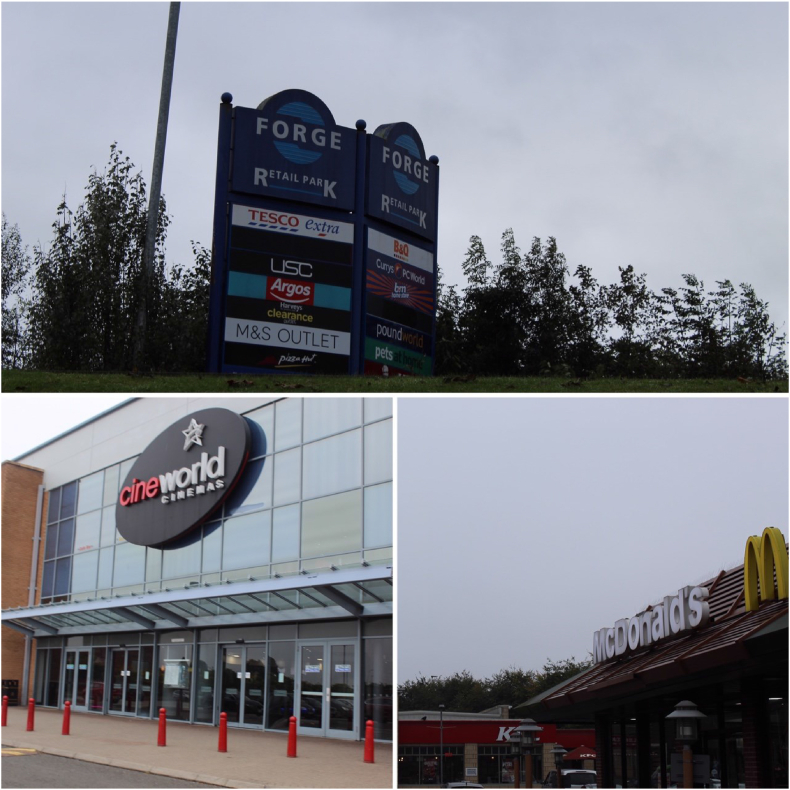
a The local shopping centre. b The cinema. c Fast food.

Reflections on boredom from the participants relate to a perceived lack of resources in the neighbourhood. They observed that there was very little for children or young people to do on their own without adult supervision. Whilst they reported enjoyment from the open spaces of parks, they recognise that they are *“not really hangout places”.* They gave examples of facilities that they would like in these spaces, such as football pitches and bike trails. In contrast, the participants discussed elements of the built environment as accessible to them without adult supervision, spaces where they could *“hang out”* (Fig. 6a). Their ability to take part in activities in these spaces was however limited both by availability and cost. The young researchers discussed how there were activities for young children (soft play) and for older children/young adults (arcades and gambling), but felt that their age group was not catered for. They noted that there was “*not enough to do”,* that they were *“bored of doing the same things”* and that they could “*end up depressed”,* making the connection between the lack of facilities in their neighbourhood and their mental well-being. The cinema or ice rink may be accessible to them, but it is too expensive for many to go there regularly with family or friends: *“you need money to come here”* (Fig. 6b). The young researchers recognised the inequalities that arise as a result, stating that “*people could have to miss out or not be able to do things if parents can't give them money”*. They discussed how some children will have these resources available to them, so the lack of free activities would be irrelevant, but for others these activities are inaccessible. The participants suggested that to reduce these inequalities, *“a range of other free/cheap activities with lots of opportunities and options”* are required. They gave examples such as community centres and youth clubs.

One activity that was available to them and that was cited often by the participants in both areas was eating out. Whilst some of the participants remarked that this too was expensive, they did recognise that the many fast-food restaurants (Fig. 6c) in their neighbourhoods were somewhere they could go to socialise, places to eat that *“might be more affordable for families”* or that are *“cheap,* [with] *young people more likely to go there”*. In health terms, the children recognised that such fast-food establishments were not healthy, but that the *“food tastes good, even though [you] know it's not good”*. It was noted that “*most of the food options are unhealthy”* and that the restricted range of options meant that there was little alternative. The participants, particularly those of high school age, did however view these spaces as positive resources for them to draw on, reflecting the lack of alternative activities available for them in the neighbourhoods.

## 4 Discussion

This paper makes a timely contribution to the literature focussed on children and young people's sense of place, and in particular, their understanding of the connections between environment and health. While we know that the local environment and neighbourhood can impact upon the health of children and young people, we know far less about how children and young people perceive this relationship. Focussing on two income deprived urban neighbourhoods in Scotland, this article has explored children and young people's perceptions of how features of their neighbourhoods can shape their health and well-being and contribute to health inequalities. The project was designed to ensure meaningful and active participation of children and young people; from the design of the research themes and the selection of methods through to the analysis, the young researchers led the work at all stages. The themes explored by the young researchers of safety, littering, and friends and family shaped the focus groups, mapping exercise and community walks. In the analysis of this material by the young researchers, it became clear that children and young people have a well-established understanding of how their neighbourhoods impact upon their health and well-being. Less well-established was their consideration or understanding of inequalities, but some evidence did arise. From a review of the young researchers' analysis, it is also possible to identify some overarching concerns of stigma, exclusion and trust.

The participants conveyed a sense of exclusion from the places and spaces they experience on a regular basis. This exclusion resulted from a complex interplay of personal encounters, financial barriers, public attitudes and limited child-friendly resources. In particular, a lack of financial resources was emphasised, with this and contextual barriers converging to construct spaces where children experience exclusion. Related to this, the participants reported feelings of boredom, depression, and unfairness. The children and young people in this study wanted to take part in shared activities, but identified that financial barriers could reduce their ability to do so, particularly in areas where there are few free alternatives available. Our findings reveal, from the perspectives of children, how disadvantage can restrict their everyday childhood experiences (Ridge, 2011) and exclude them from private services (Wager et al., 2007). The importance of having alternative spaces where children and young people can spend time has been identified in previous research (Carroll et al., 2015; Witten et al., 2015). Such spaces have been referred to as ‘third places’, distinguishing them from the confines of the ‘first place’ (home) and ‘second place’ (school) (Carroll et al., 2015; Witten et al., 2015). These spaces can provide children and young people with opportunities to interact and meet with others with exclusion resulting in children and young people “missing out on the social, physical and cognitive advantages of independent mobility” (Witten et al., 2015, p. 354).

The exclusion experienced by the children and young people from these ‘third places’ may limit their opportunities for vital play and development. In our study, this exclusion extended to private spaces (e.g. shopping centres and high streets) and public, open spaces which were seen as unsafe and run down, echoing previous work where young people identified areas to be avoided (Chawla and Malone, 2003). In the open spaces, participants reported having to negotiate large rubbish piles, discarded needles, broken glass and play equipment that was often damaged and degraded. Many reported that this would affect how much they wanted to use these spaces to play and socialise. More populated spaces within the built environment, such as high streets, were also seen as unsafe, particularly related to substance misuse and intoxicated adults, reflecting similar findings from the Scottish Children's Parliament (Alcohol Focus Scotland and the Children's Parliament, 2019). This sense of exclusion and vulnerability in public space has led some to ask whether spaces that exclude and marginalise young people can be described as ‘public’ (Valentine, 2004). The retrenchment of local spending during the UK's period of austerity may have amplified the experiences of these children and young people. Pearce (2013) argues that residents in middle class areas are better able to resist the cuts in services with lower income neighbourhoods therefore disproportionately affected. The result may be that many children are left anxious about the lack of money and resources available to them (Ridge, 2009).

The narrative running throughout our discussions with the children and young people was that they face a range of barriers to participation in society, exacerbated by a lack of affordable alternatives. The participants reported related unhappiness and anxiety reflecting similar findings elsewhere (Crowley and Vulliamy, 2007; Ridge, 2002). Such experiences limit young people's socio-spatial mobilities and constrain low-income children to their local neighbourhoods (Wager et al., 2007). The increasing commodification of childhood experiences may therefore have the most severe impact on the health and wellbeing of disadvantaged families (Ridge, 2013). Such children require safe, free, outdoor spaces to roam as alternatives to the more expensive leisure facilities that they are unable to access (Elsley, 2004).

In addition to exclusion, place-based stigma was also experienced. Whilst the participants did not mention experiencing any explicit discrimination, stereotyping or labelling (as identified by Link and Phelan (2001)), they did discuss an internalised stigma and resistance to it that reflects arguments reminiscent of Waquant (2008). Halliday et al. (2021) have summarised distinct pathways between spatial stigma and health with two of these clearly identified in this analysis. First, spatial stigma may cause psychological distress, with feelings of shame linked to adverse mental health outcomes. Reflecting this, the participants were conscious of negative features of their neighbourhoods, for example large amounts of litter, abandoned buildings, discarded needles and broken glass, and how others may then perceive them and their neighbourhood. They noted how the related shame would deter them from inviting friends over, thus diminishing their social space even further. Reflecting work by Keene and Padilla (2010), the participants were aware that the area reputation can also shape their own feelings towards the neighbourhood. The second pathway highlighted here relates to ‘the psychosocial impact of moral inferiority’ (Halliday, 2021). The participants expressed an awareness of differences between the ‘posh’ neighbourhoods and their own. They were acutely aware that these differences would mean that the opportunities afforded to those in the wealthier neighbourhoods would be significantly different to their own. The participants discussed feelings of being ‘looked down on’, with evidence suggesting that such feelings are detrimental to life chances (Pearce, 2013). This internalised stigma was reflected in the discussion of fences and trusted adults and the perceived need for increased policing and security. The children's sense of abandonment, reflected in broken play equipment, litter and the lack of resources, underpinned the spatial comparisons that they made between neighbourhoods of varying affluence. The participants connected these inequalities and the reputation of the area to their own health and well-being, including stress and anxiety. Keene and Padilla (2014) have recognised this mechanism acknowledging that “disadvantaged places contribute to multiple physical and mental health outcomes” (p. 392), whilst Halliday et al. (2020) have called for greater public health attention to spatial stigmas as a public health concern. Our paper demonstrates that children and young people can feel this stigma, and the socio-spatial polarisation that results may impact negatively on their health and well-being. Some of the responses from the children show how stigma is interwoven into their experiences of place. Despite these negative experiences, support from family and friends, and the resulting social cohesion, enables children and young people to use the space in positive ways, resisting the place-based stigma that may constrict their use of space (Thomas, 2016).

Although much of the children and young people's narratives were characterised by negative experiences of place, their experiences were not universal, emphasising that there is no “single sense of place that everyone shares” (Massey, D, 1993, p. 60). Whilst the participants revealed similar experiences of the neighbourhoods, classification of environmental resources as either ‘good’ or ‘bad’ masks the uneven perceptions held by the children and may be “unduly naïve and simplistic” (Macintyre, 2007, p. 5). This highlights the complex geography of belonging situated within the children's narratives. Within this complexity and the heterogeneity of the participants experiences exists also a sense of place that exudes more positive connections. The children's sense of belonging was apparent and crucial to this was the role of trust. To them, trust, particularly trust in adults, enabled them to ‘belong’, to use spaces which may at first appear to be unsafe or dangerous. Such feelings of neighbourhood trust have strong associations with children's psychopathology (a measure of mental health in children) measured through survey instruments (Meltzer et al., 2007), and in our study, trust invoked a sense of belonging, and in turn, well-being. Their sense of familiarity and comfort in their local neighbourhoods was supported by the network of connections existing to them. Trust in adults was related to positive feelings of safety and inclusion despite the difficulties they experience in other areas.

In this project, we followed a child centred approach to project design, methodology and interpretation. Our research responds to an increasing recognition that “children are rarely recognised in neighbourhood research and their perceptions of the neighborhoods and the environments they occupy every day go largely unnoticed” (Schaefer-McDaniel, 2009, p. 417). However, the approach of ‘putting children first’ and enabling children and young people in research has been gaining traction, and as such, our understanding of child-friendly spaces is improving (Bradbury-Jones and Taylor, 2015; Carroll et al., 2015; Ergler et al., 2021). It must however be recognised that there were limitations to our approach. The method applied to the research meant that the determinants of health that were shown to the young researchers through the flash cards were defined by adults from topics identified in the literature. The young researchers then selected the topics they found most important, and whilst they had the opportunity to lead discussion and identify new topics, they did not do so. It would be of value to explore how young researchers could identify new determinants of health in future research. However, it is important to consider how this would affect the delivery of such projects. Having a clear framework for the research supported the researchers to engage with the project and to develop their thinking. In a model of co-production, the adults involved in the project were able to learn from the children and the children themselves gained an experience that they relished. At the end of the project the young researchers reflected on their time spent on the research with one stating that *“it was not as boring as they thought and there were ways of doing it that made it fun”*. They told us how the project had been their first chance to think about where they lived in a more critical way. Some said it had opened their eyes to the area that they lived in and that they were now more aware of their surroundings. Other benefits of participation noted by the researchers was the opportunity to make new friends and being more confident to speak up and share their views.

Much of the research on children's experiences of environment and health has relied on parental descriptions of childhood experiences (Reay and Lucey, 2000). Our findings offer important starting points for future research with children and young people. Children's experiences of place are layered and multi-faceted, impacting not only their use of space in the present, but potentially into the future as they transition into adulthood. Our findings on child-centred conceptualisations of stigma and exclusion offer critical insights for future work on stigma as a fundamental determinant of health and health inequalities. Future work could critically examine the role of stigma and exclusion in specific health outcomes. In particular, there is a need to consider the role of place-based stigma in the construction of health-related behaviours in children and adolescents; such behaviours were referred to in this paper but not explored in detail. Pearce, Barnett and Moon (2011) discuss how some residents of socially deprived neighbourhoods may be subject to dual stigmatisation (such as smoking and residing in a low-income area), but more work is needed on how stigma itself may play a role in forming health behaviours as social practices in childhood and adolescence (Frohlich et al., 2002). This role of stigma and exclusion in creating the social structure of context may add to our understanding of related inequalities.

Whilst previous research has highlighted the ways in which children and young people can be consulted in policy formation, a review by Sullivan et al. demonstrated how “few concrete policies or documents existed related to child consultation” (2021, p. 37). In a review of child friendly planning around the UK, Wood et al. (2019) set out recommendations to make positive change in the planning system in order to realise children's rights. These recommendations include the need for children's mobility and independence to be given prominence in planning decisions (reflecting the earlier discussion of the use of ‘Third Places’), and the need for national policy stipulating that children have a right to be included in planning decision-making. Sutton (2008) has argued that “government policy is most frequently concerned with improving deprived children's futures, rather than their experiences as children” (p. 546). It is from this experience of the ‘here and now’ of childhood from which the young researchers in this project have drawn a list of recommendations. These recommendations reflect those discussed by Wood et al. (2019) and are related to children and young people's sense of place and daily experiences and practices. The recommendations for action in this area represent a shift from a deficit perspective to one of inclusion and belonging. First, the young researchers would like more visible responsible adults in their neighbourhoods (such as police or community wardens) to help them feel more safe and secure. Second, they would like better access to free or cheap activities, reflecting the discussion on exclusion. Third, recognising the importance of open space, they would like the quality of green spaces to be improved, broken play equipment fixed, litter collected, abandoned spaces regenerated and vandalism dealt with. Fourth, the young researchers would like more support for those in their neighbourhood with substance misuse problems, stating that they want “support not stigma”, and opportunities to build positive relationships in the community. Fifth, they would like the planning process to acknowledge the abundance of unhealthy retailers in their neighbourhood with greater support for healthier shops and restaurants. Related to this, they would also like free bus travel to enable them to access such resources that may be outside of their immediate vicinity. Finally, the young researchers want to be heard. They want to be involved in decision making “about the places we live. This is our right”.

## Author contributions and acknowledgements

CR conceived of the study, acquired the funding and led the project administration. CR and NKS were involved in the research, drafted and edited the paper. The authors would like to thank the young peer researchers from both schools. This paper is based on their findings and their work has added a wealth of new evidence. We also must thank all the children and young people who participated in the research. Thank you also to colleagues at Children in Scotland who were involved in supporting the delivery of the initial project and in disseminating the findings. This research was funded by the Wellcome Trust, grant reference number: 214801/Z/18/ZThe Wellcome Trust were not involved in the development of the project.
